# Face the Hierarchy: ERP and Oscillatory Brain Responses in Social Rank Processing

**DOI:** 10.1371/journal.pone.0091451

**Published:** 2014-03-12

**Authors:** Audrey Breton, Karim Jerbi, Marie-Anne Henaff, Anne Cheylus, Jean-Yves Baudouin, Christina Schmitz, Pierre Krolak-Salmon, Jean-Baptiste Van der Henst

**Affiliations:** 1 CNRS, Laboratoire Langage, Cerveau et Cognition (L2C2), Université Lyon 1, UMR 5304, Bron, France; 2 CNRS/INSERM, Centre de Recherche en Neuroscience de Lyon (CNRL), U1028, UMR5292, Bron, France; 3 Université de Bourgogne, Pôle AAFE, Dijon, France; Vanderbilt University, United States of America

## Abstract

Recognition of social hierarchy is a key feature that helps us navigate through our complex social environment. Neuroimaging studies have identified brain structures involved in the processing of hierarchical stimuli but the precise temporal dynamics of brain activity associated with such processing remains largely unknown. Here, we used electroencephalography to examine the effect of social hierarchy on neural responses elicited by faces. In contrast to previous studies, the key manipulation was that a hierarchical context was constructed, not by varying facial expressions, but by presenting neutral-expression faces in a game setting. Once the performance-based hierarchy was established, participants were presented with high-rank, middle-rank and low-rank player faces and had to evaluate the rank of each face with respect to their own position. Both event-related potentials and task-related oscillatory activity were investigated. Three main findings emerge from the study. First, the experimental manipulation had no effect on the early N170 component, which may suggest that hierarchy did not modulate the structural encoding of neutral-expression faces. Second, hierarchy significantly modulated the amplitude of the late positive potential (LPP) within a 400–700 ms time-window, with more a prominent LPP occurring when the participants processed the face of the highest-rank player. Third, high-rank faces were associated with the highest reduction of alpha power. Taken together these findings provide novel electrophysiological evidence for enhanced allocation of attentional resource in the presence of high-rank faces. At a broader level, this study brings new insights into the neural processing underlying social categorization.

## Introduction

Living in a sophisticated social environment is cognitively more demanding than living alone. According to the social brain hypothesis, this demand exerts selective pressure on the brain of social species [1]. A recurring feature that contributes to the complexity of social environments is *hierarchy*. A large proportion of primates' social relationships are indeed asymmetrical: some individuals have priority of access to resources, some are endowed with greater social prestige, and some exert control over others. Humans are no exception: although the hierarchical organization varies among cultures, all current and past societies have shown some degree of stratification [2], [3] and developmental studies have reported that children as young as two-year old form stable hierarchies [4]. The evolutionary importance of hierarchy manifests itself through its consequences on reproductive success since high-rank individuals tend to have more offspring than low-rank individuals [5], [6].

In order to successfully navigate their social environment, humans need to categorize individuals according to their status. Behavioral studies indicate that adults and even infants display a remarkable ability to recognize social asymmetries [7], [8]. At the brain level, although recent fMRI studies have reported a wide range of brain structures involved in the processing of hierarchical stimuli, such as the medial, dorsolateral and ventrolateral prefrontal cortices [9], [10], [11], the amygdala and anterior hippocampus [12], the temporal dynamics of the neural response associated with such processing remains unknown. When does the neural response elicited by the hierarchical nature of stimuli emerge and what is the nature of the rapid subprocesses elicited by those stimuli? To address these questions we used electroencephalography (EEG) and investigate the neural mechanisms involved in processing *faces* that were embedded in a learned hierarchical context. Faces were used as stimuli because they are highly relevant to socio-cognitive processes and can therefore be easily linked with social aspects of the environment. However, in order to avoid any confound with physical modification of the face [13], the manipulation of social rank should not be associated with any specific facial expressions. Consequently, the key manipulation consisted of presenting faces with *neutral* expressions in a game context where the subject had to rank other players performance with respect to his own.

Identifying one person's rank is a form of social categorization as it consists of associating an individual with scalar social categories (High-status>Intermediate-status>Low-status). Previous EEG studies have mainly investigated the social categorization of faces through dichotomic categories such as race (i.e. racial ingroup vs. racial outgroup faces), gender (female vs. male faces) or familiarity (familiar vs. stranger faces). A critical issue was whether these categories modulate the amplitude of the well-known face-related N170 component, which is thought to depict an early stage of processing associated with the structural encoding of faces ([14], see [15] for a review). Many studies report that the N170 component is insensitive to social categories (familiarity: [16]–[22], gender: [23], [24], race: [25]–[27],) and suggest that social information conveyed by a face is processed only after its structural encoding. This sequential view has been challenged by a significant number of studies that did observe an influence of social categories on the electric N170 or magnetic M170 (familiarity: [28]–[33], gender: [13], race: [34]–[36]). Interestingly, one of the only EEG investigations which has explored facial expressions related to hierarchy reported that facial expressions of dominance elicited a N170-like component whose amplitude was greater for facial expressions of submission [37]. However, this study did not only manipulate hierarchy as physical properties of faces were also varied. Taken together, these results suggest that whether the N170 is modulated by hierarchy is still an open question.

While the aforementioned N170 findings are partly inconsistent, the literature concurs that social categorization modulates the Late Positive Potential (LPP), revealing an influence of higher-order cognitive processes. The LPP associated with familiar faces has been interpreted as the activation of semantic and personal identity representations [18], [20], [38], while those elicited by race and gender have been interpreted as the cognitive assessment of face and category-related information [35], [39] or as the manifestation of evaluative social judgments [27]. In light of these results, it could be expected that hierarchy, being a major feature of social categorization, would modulate the LPP elicited by faces. To the best of our knowledge, there is so far no clear evidence for this hypothesis in the literature.

Hence, the first goal of this study is to determine whether hierarchy influences the early structural processing of faces or/and whether it influences a higher-order processing stage. In addition, the second goal of this study is to further explore the cortical mechanisms related to hierarchical face processing by investigating the putative involvement of neural oscillations. A parallel stream of research has shown that the perception of faces has been associated with suppressions in alpha-band power (9–12 Hz) in the occipito-parietal areas [40], [41]. However, whether such oscillatory power modulations are differentially affected by social rank remains unknown.

In the current experiment, the social hierarchy was learned through direct experience in a competitive game. This procedure differs from previous fMRI studies in which participants did not observe the building of the hierarchy but were directly provided with status information through perceptual cues, such as body posture [10], uniforms [42], stars [9], or through famous individuals [11]. The only brain study where hierarchy was acquired through experience is that of Kumaran et al. [12] in which participants learned the status of individuals on the basis of a transitive reasoning task. This type of task is often used in ethology in order to show that the verticality of hierarchical relations tends to shape the inferential abilities of social species [43]–[46]. Typically, those tasks involve a wide range of pairwise relations (A>B; B>C; C>D; D>E; E>F; F>G) from which new relations can be drawn (i.e. B>E) and from which the entire hierarchy can be built (A>B>C>D>E>F>G). Here, we used a simpler procedure in which participants were directly provided with the whole ranking of four players. This is a relatively ecological procedure as in a wide range of human competitive activities the full hierarchy is communicated and does not need to be inferred from adjacent dyads. University rankings, sports rankings, the Forbes world's billionaires list are typical examples. Moreover, transitivity tasks are used when the hierarchy is fully vertical but this was not the case in the present task as two players had the same rank (i.e. the participant and one of the other players, see below).

## Materials and Methods

### Ethics Statement

All participants gave written informed consent for the study which was approved by the local Ethical Committee (Comité de Protection des Personnes, Gerling agency- File no: 90788200730). All participants consented to participate in the research on their own behalf. Prior to the task, the experimenters made sure that participants had the capacity to consent by asking a series of questions about their understanding of the study.

### Participants

Sixteen right-handed healthy male volunteers aged between 19 and 27 years (mean  =  21.05 years ± 1.6 SD, median  =  20.96 years) participated in this study. All participants were French, had comparable educational backgrounds and had normal or corrected-to-normal vision. To avoid possible confounds from gender-related differences in sensitivity to competitive settings [47] only male volunteers were tested, and they were confronted only with male faces. Participants were compensated for their participation.

### Stimuli

The stimulus set consisted of 36 high-resolution photographs of neutral male faces picked from the NimStim Set of Facial Expressions standardized database [48] and from the stimuli set used by Baudouin and Gallay [49]. No remarkable details, such as glasses, earrings, piercings or mustaches, were visible on the faces. All pictures were transformed into grayscale images and normalized in size, luminance and contrast. They were displayed on a screen with a light grey background and subtended a horizontal visual angle of 9.8° and a vertical angle of 7.7° at a viewing distance of one meter (480 × 374 pixels).

### Design and Procedure

**Figure 1:**
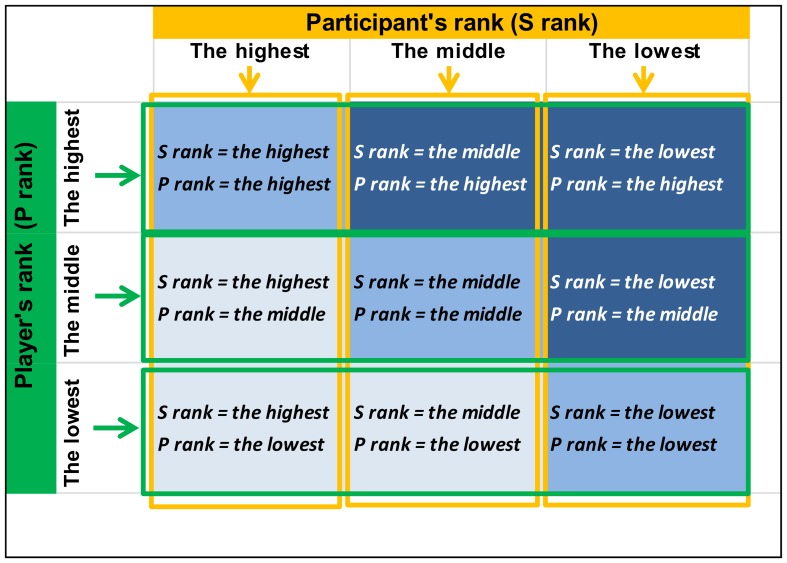
Representation of the nine experimental sub-conditions in the design. Columns (yellow) correspond to the three participant rank conditions (highest, middle and lowest rank); rows (green) depict the three player rank conditions (the same three conditions). Note that the term “*Player*” refers to the face stimuli, while the participant is the subject that took part in the EEG experiment.

Upon his arrival to the laboratory, the participant's face was photographed and inserted into the group of faces as a landmark of his position. We created a competitive context to ensure that the participant really believed he was included in a hierarchy. As a cover story, he was told that he was participating in the second part of a research project conducted on network games and visual perception. In the first part, other volunteers supposedly already completed the same games that the participant would play, and their scores and pictures had been stored in the database. For each game, the participant was told that his performance would be compared to 3 “former players”, represented by their photographs, and randomly chosen from the database. Participant was told that these “former players” have been previously ranked according to their scores as the best, middle or worst competitor. Participants were thus presented with faces of three ranks corresponding to three **player rank** conditions: **highest, middle** and **lowest rank**. Additional filler stimuli included faces referring to players whose ranks were unknown. Participants were provided with information regarding their own status, which could be equal to the highest, middle or lowest rank. The ranks of the supposed players and of the participant were thus manipulated in a systematic way, giving rise to a 3-by-3 within-subject design (Figure 1). In each block, participants saw his face and four other faces, corresponding to the three rank conditions (i.e., highest, middle and lowest) and one filler face whose rank was unknown. Four different faces were used in each block so that a given face never occurred in two different blocks. Participant rank was manipulated across blocks and not within each block: in three blocks, the participant had the highest rank (two players were lower than him and one equal to him). In three other blocks, he was in the middle position (one player higher, one lower and one equal to him) and in three blocks, he had the lowest rank (two players were higher and one equal to him). Thus, the three **participant rank** conditions were **highest**, **middle** or **lowest**. The order of these blocks was set randomly across participants. Moreover the presentation of faces was counterbalanced across participants so that each face was equiprobably associated with each possible rank.

The participant was comfortably seated inside a dimly lit Faraday cage in front of a screen. He first performed a training session constructed as an experimental block. The experiment was designed using Presentation 14.3 (Neurobehavioral System, http://www.neurobs.com/) and was divided into nine blocks, including the nine different game sequences. Each experimental block was designed to adhere to the following sequence: a game phase that served to contextualize faces within a hierarchy and manipulate participant and player ranks (Phase 1), a ranking restitution phase (Phase 2), and a face rank evaluation phase (Phase 3). Each participant took part in the three phases. EEG was only analyzed in the third phase. After the last phase was completed, the participant was instructed to start the next block.

#### Phase 1: game phase

**Figure 2:**
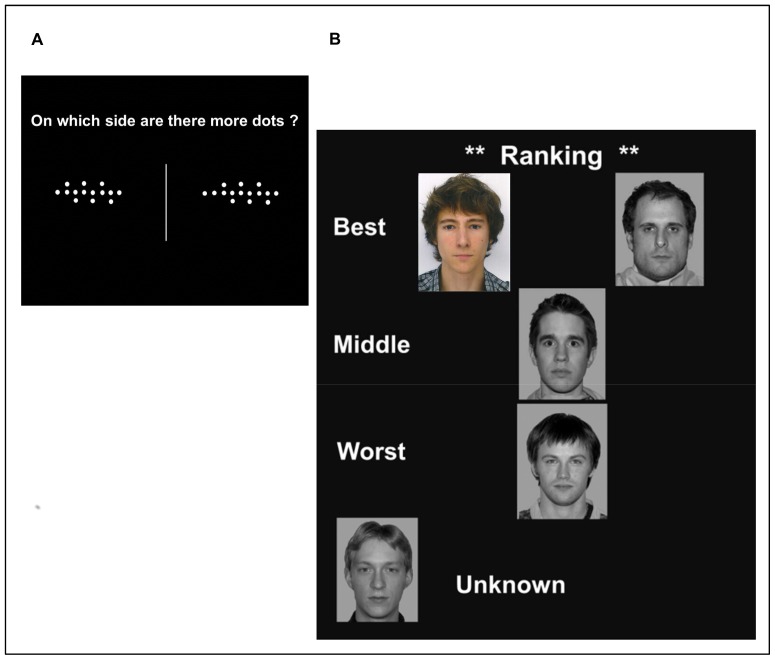
Examples of round and associated ranking proposed to the participant during phase 1. A) Example of one round of the game during phase 1. In this game, the participant was asked to decide which side of the screen contained the largest amount of dots. B) Example of three-level ranking presented to the participant after each round of the game. Participant's face was the only photograph displayed in color during this phase. All the subjects of the photographs used here have given written informed consent, as outlined in the PLOS consent form, to publication of their photograph.

A series of five simple perceptive tasks (or rounds) was presented to the participant, such as detecting which side of the screen contained a greater amount of dots as fast as possible (Figure 2A). The participant was told that his performance in each of the five tasks, allegedly based on reaction time and validity of the answers, would be compared to three other players who completed the same tasks. This comparison gave rise to an intermediate ranking presented after each task, in which one player had the same rank as the participant and two other players had a different rank. Faces, representing players, were displayed vertically on three hierarchical levels labeled, from top to bottom, as the “best” player, the “middle” player and the “worst” player. The participant's photograph was also inserted in the ranking (Figure 2B). The “**no rank**” player face appeared in the bottom left corner of the screen. The participant was told that the rankings were established by a cumulative score of the player's performances on all tasks. For example, the participant's rank after the second task was claimed to be determined by the averaged performance over the two first tasks, and so on until the final ranking following the fifth task. Real performances were not taken into account to rank the participant. Intermediate rankings were fixed according to the final intended ranking. Four of the 5 rankings were identical to the intended one, and in one task randomly chosen among the first three, the participant's rank differed by one position in the hierarchy. This procedure was applied for maintaining credibility. Participant's rank was thus fully established during the fourth and last rankings in Phase 1 and did not change over Phase 2 and Phase 3. Participant rank could only change in the next block when starting a new game phase (i.e. Phase 1).

#### Phase 2: ranking restitution phase

At the end of the game, participants were asked to reproduce the cumulative ranking established over the 5 tasks in Phase 1. To do so, they had to use the mouse and move the 5 photographs (those of the four players and their own photograph), which were horizontally displayed at the bottom of the screen. If the ranking was incorrect, a sixth task (never shown before) was presented before starting the third phase. This sixth task occurred only 6 times.

#### Phase 3: rank evaluation phase

**Figure 3:**
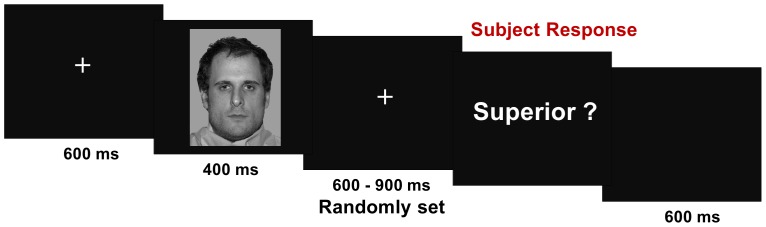
Temporal course representation of stimuli displayed during the phase 3. The subject of the photograph has given written informed consent, as outlined in the PLOS consent form, to publication of their photograph.

During the last phase comprising 60 trials per block, the EEG was recorded. In each trial, one of the four faces ranked in phase 2 was presented for 400 ms. Each face was followed by a question concerning its rank namely, “superior?” (which meant “was his rank superior to yours?”), “equal?”, “inferior?”, “unknown?”. The participant's task was to answer the question as accurately as possible by pressing one of three response keys: “yes”, “no” or “can't tell” (Figure 3). Faces were repeated 15 times each and displayed in a pseudo-randomized order, with no face appearing in two consecutives trials. The inter-stimulus interval after subject's response was randomized between 1200 and 1500 ms. Over the course of the experiment, 540 trials (36 faces presented 15 times) were presented to the participants, comprising 405 target trials (135 trials for each of the experimental conditions) and 135 fillers. The critical trials included 45 trials of player rank (highest, middle, lowest) × participant rank (highest, middle, lowest) sub-conditions; that is, 135 either by player rank condition, or by participant rank condition.

### EEG recordings

The EEG signal was recorded simultaneously from 30 electrodes attached to an Acticap (International 10–20 System, American Encephalographic Society, 1994) amplified by a BrainAmp amplifier (Brain Products, Gilching, Germany). The sampling rate was 1024 Hz through a bandwidth of 0.01–100 Hz. During the task, the reference electrode was set on the tip of the nose and was kept for off-line analysis. Two supplementary external electrodes were set on each ear lobe. The ground electrode was placed on the forehead. Vertical and horizontal eye movements (EOG) were monitored on-line by two bipolar montages. The impedance of all electrodes was kept below 5 kΩ. Using ELAN software developed by the INSERM U1028 at the Lyon Neuroscience Research Center (CRNL), Lyon, FRANCE [50] (), trials contaminated by ocular movements, blinks (EOG activity greater than 100 μv for 100 ms) or electrical artifacts (voltage changes on any scalp electrode greater than ±150 μv) were rejected off-line before averaging. After these artifact rejections, we kept a minimum of 25 valid trials for each subject for each player's rank (3) × participant's rank (3) sub-condition (max number of trials for each condition  =  44; median number of trials in each condition between 37.5 and 40).

#### ERPs analysis

The EEG signal was band-pass filtered between 0.05 and 35 Hz and segmented into temporal epochs of 1200 ms, 200 ms prior to the stimulus onset and 1000 ms post-stimulus. For each subject, segments were then averaged across the 9 hierarchy conditions depicted in Figure 1. The averaged activity from −200 ms to 0 ms pre-stimulus was used as the baseline [51]. The early P100 ERP was measured on occipital sites (O1 and O2) and the N170 ERP was measured on temporo-parietal sites (P7 and P8). Peak latency was defined as the latency of the maximum amplitude. For each participant, the average amplitude of the evoked potentials was computed within a 40 ms time-window centered on the peak latencies, i.e., 96–136 ms for P100 and 137–177 ms for N170. To examine the LPP, we selected a cluster of nine electrodes covering the frontal (F3-Fz-F4), central (C3-Cz-C4), and parietal (P3-Pz-P4) regions over the scalp. The average amplitude of the LPP was calculated for each sub-condition in the 400–700 ms time window [52].

To determine the time course associated with the neural processing of both player rank and participant rank, mean signals and latencies associated with early potentials and mean amplitude corresponding to late potentials were submitted to distinct repeated measure ANOVAs. For early components, the ANOVA used ranks of players (3 levels: highest, middle and lowest), ranks of participants (the same 3 levels) and electrodes of interest (2 levels) as subject factors. For late components, the ANOVA tested the topography of the ERP waves by including, instead of the electrodes, the spatial parameters caudality (frontal, central and parietal) and laterality (left, midline and right) as supplementary within subject factors. The effect of relative hierarchical status (Superior, Equal or Inferior) on the perception of faces was studied by examining the interaction between face and participant ranks. Least significant difference (LSD) Fischer tests were used for post-hoc comparisons.

#### Time-frequency analysis

The non-filtered EEG signal was analyzed in the time-frequency (TF) domain by convolution with complex Gaussian Morlet's wavelets [53]. This analysis enabled us to explore spectral power variations generated by the perception of faces embedded in different hierarchical contexts. Based on previous reports of alpha-range modulations in face perception [41], we specifically focused the TF assessment of task-related power changes to the alpha-band (9–12 Hz). After artifact rejections, data were segmented and averaged for each sub-condition using [−300; −50 ms] as the baseline interval. This interval was chosen to avoid smearing artifacts generated by computing wavelets and to include at least two periods of the oscillatory response (at 10 Hz, 200 ms are needed for achieving two oscillatory cycles). The same Elan software was used to display TF maps on the [−300–1000 ms] × [1–90 Hz] domain. First, we evaluated absolute power variations of alpha rhythms associated with face perception defined as energy increases or decreases relative to the energy level prior to face presentation. In each of the nine sub-conditions, the energy in the [9–12 Hz] × [0–1000 ms] domain was compared with a tile of the same frequency extent chosen in its respective baseline period [−300;−50 ms], using Wilcoxon matched paired nonparametric tests. The result of a Wilcoxon test is a Z score that indexes the spectral power difference between two conditions. This score was used to generate statistical TF maps representing spectral energy modulations across time and frequency bands for each electrode. Associated with the Z score, the p value was represented in topographic maps. These maps allowed us to identify temporal intervals and scalp regions supporting statistically significant variations (i.e., *p*<0.05) compared to the baseline. Second, to detect relative differences in the alpha oscillatory response depending on player or/and participant ranks, the same statistical analysis conducted for evoked potential was performed on TF signal. According to the Wilcoxon results, we delineated a 300 ms temporal window in which the alpha power statistically decreased compared to the baseline. Based on the averaged frequency profile between 9 and 12 Hz, the mean alpha power was computed on the [400 ms–700 ms] interval in each sub-condition. We selected the same cluster of 9 electrodes described above (F3, Fz, F4, C3, Cz, C4, P3, Pz and P4) and we compared the alpha mean responses over this temporal interval using an ANOVA including the topographic variables caudality and laterality (3 levels each – see above), the ranks of players (3 levels: highest, middle and lowest) and the ranks of participants (the same 3 levels). Post-hoc analysis consisted of LSD Fisher's tests.

## Results

### Behavioral data

Participants were very accurate in categorizing player ranks, obtaining more than 95% correct responses (mean number of misses ± SE  =  27 ± 30.1 among 540 trials, minimum number of misses  =  5, maximum number  =  105).

### Electrophysiological data

#### P100

**Figure 4:**
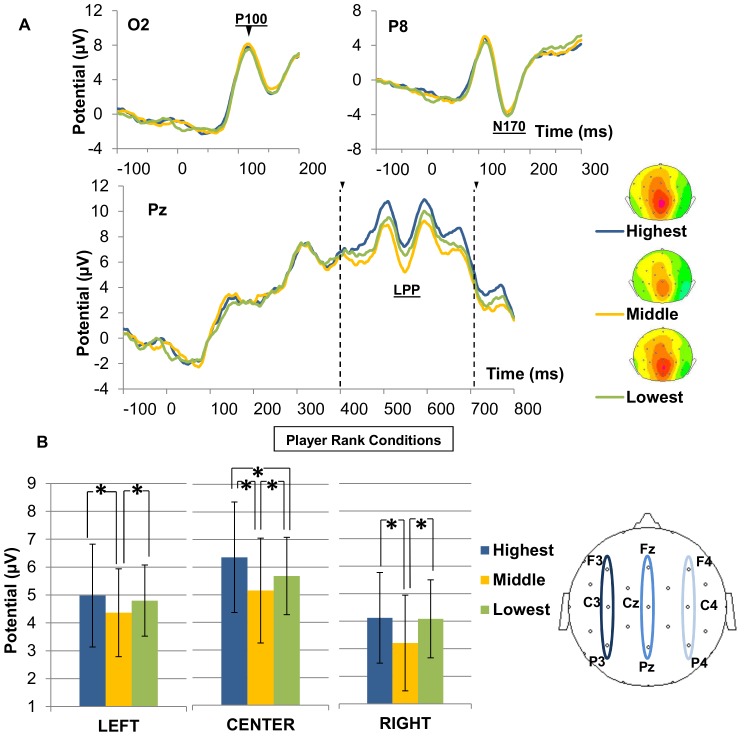
ERP waveforms and averaged signal of P100, N170 and LPP components. A) ERP waveforms of early (P100 and N170) and late (LPP) responses in the three player rank conditions (highest, middle or lowest rank). Associated back-view topographic maps represent the averaged amplitude of LLPs at 550 ms. B) Averaged LPP amplitude in the 400–700 ms time window computed on the left (F3, C3, P3), central (FZ, CZ, PZ) and right (F4, C4, P4) scalp regions in the 3 player rank conditions. * indicates a significant difference (p<0.05).

The P100 showed a mean latency of 116 ms after face onset (Figure 4A, top left). The amplitude of this potential was neither subject to a main effect of electrodes (F(1,15)  =  0,09; *p*  =  0.76) nor to participant rank (F(2,30)  =  0,41; *p*  =  0.66) nor to player rank (F(2,30)  =  0,81; *p*  =  0.45). No participant rank × player rank interaction was observed (F(4,60) = 0,48; *p*  =  0.74) and the triple interaction was not significant (F(4,60)  =  0,47; *p*  =  0.75).

#### N170

The N170 component reached its maximal amplitude at 157 ms (Figure 4A, top-right). The ANOVA revealed that the N170 amplitude was neither sensitive to hemisphere (F(1,15)  =  0,96; *p*  =  0.34), nor to participant rank (F(2,30)  =  0,45; *p*  =  0.63), nor to player rank (F(2,30)  =  0,87; *p*  =  0.42). Moreover, there was no player rank × participant rank interaction (F(4,60)  =  0,51; *p*  =  0.72), and no player rank × participant rank × hemisphere interaction (F(4,60)  =  0,94; *p*  =  0.44). Moreover, the N170 latency was neither sensitive to hemisphere (F(1,15)  =  1,51; *p*  =  0.23), nor to participant rank (F(2,30)  =  1,13; *p*  =  0.33), nor to player rank (F(2,30)  =  0,60; *p*  =  0.55), and there was no significant interaction observed.

#### Late Positive Potential

As shown in Figure 4, we also observed the LPP between 400 and 700 ms over nine recording sites (Table 1 – Figure 4A, bottom). The ANOVA revealed a main effect of caudality (F(2,30)  =  25,81; *p* < 0.001). The LPP followed a frontal < central < parietal amplitude gradient, consistent with the scalp distribution described in the literature [54]. The ANOVA also showed a main effect of laterality (F(2,30)  =  18,18; *p* < 0.001). Post-hoc LSD tests indicated that the LPP was of greater amplitude over midline regions compared to left (*p* < 0.01) or right domains (*p* < 0.001) and larger over left regions compared to right regions (*p* < 0.01). Caudality and laterality significantly interacted (F(4,60)  =  3,75; *p* < 0.01) indicating that the laterality pattern was the most pronounced at parietal sites.

**Table 1:** Mean (± *SE*) LPP amplitude (in Microvolts; 400–700 msec) in the for the Highest, Middle and Lowest player as a function of the scalp domain (left, central and right).

	Player's rank							
	Highest		Middle		Lowest		Total	
**Scalp Domain**	M	*SE*	M	*SE*	M	*SE*	M	*SE*
Left	4,98	3,76	4,36	3,21	4,80	2,60	4,71	3,17
Central	6,36	4,08	5,15	3,87	5,68	2,85	5,73	3,60
Right	4,12	3,36	3,21	3,54	4,09	2,89	3,81	3,23
**Total**	**5,15**	**3,78**	**4,24**	**3,57**	**4,86**	**2,80**	**4,75**	**3,41**

Neither a main effect of player rank nor of participant rank was observed. However, the ANOVA showed a significant interaction between laterality and player rank (F(4,60)  =  4,12; *p* < 0.01), suggesting that the neuronal response profile to hierarchical rank varied across scalp locations (Figure 4B). In the midline domain, where the LPP was the most prominent, post-hoc LSD tests showed differences between the player rank conditions. Processing highest-rank faces elicited the largest LPP, compared to the middle-rank faces (*p* < 0.001) or the lowest-rank faces (*p* < 0.001). Furthermore, the LPP generated by the lowest-rank faces was larger than the LPP generated by the middle-rank faces (*p* < 0.01). We ensured that this averaged LPP pattern in the midline domain was observed at the subject-level by plotting for each subject the pairwise comparison of LPP amplitude in the three players rank conditions (see Figure S1). In the left and right lateralized regions, the LPP evoked by the middle-rank faces was significantly lower than this evoked by the highest-rank faces (left-lateralized sites: *p* < 0.001, right-lateralized sites: *p* < 0.001) or by lowest-rank faces (left-lateralized sites: *p* < 0.05, right-lateralized sites: *p* < 0.001), but there was no significant difference between these two hierarchical ranks (left-lateralized sites: *p*  =  0.1, right-lateralized sites: *p*  =  0.68). Finally, there was no interaction between player and participant rank (F(4,60)  =  1,02; *p*  =  0.4).

### Oscillatory components: Alpha rhythms

**Figure 5:**
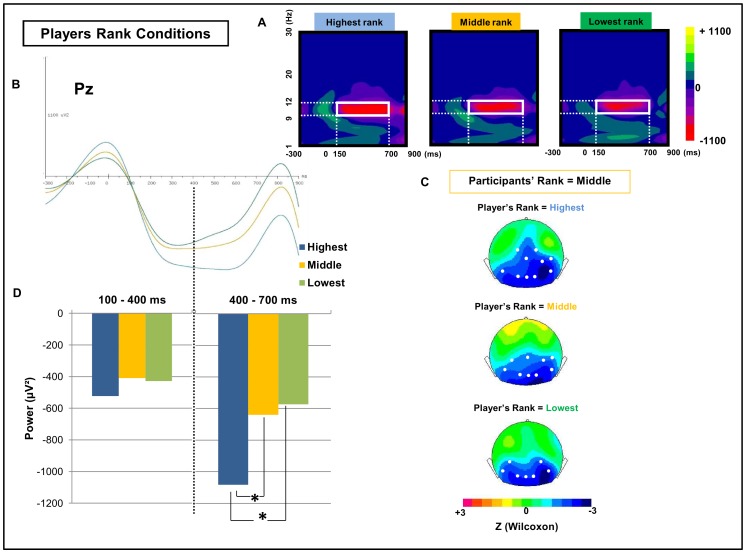
TF maps and averaged profile of alpha oscillatory response. A) TF map depicting the color-coded mean energy (in μV^2^) in response to faces in the three player rank conditions (Highest, Middle and Lowest) at the electrode Pz. The red zone depicts an energy decrease in the [9–12 Hz] × [150–700 ms] TF domain (white box). B) Time course of the energy in the alpha band during the responses to the three player rank conditions: highest (blue), middle (yellow) and lowest (green). C) Z-score topographic maps for the Wilcoxon comparisons with the baseline when the participant was in the middle position, in the three sub-conditions of player rank. The player's relative status is, from top to bottom, superior, equal and inferior. White dots indicate electrodes where the alpha reduction was significant compared with the baseline (p<0.05). D) Averaged alpha power for the three player rank conditions computed on Pz in [100–400 ms] and [400–700 ms]. Significant differences (p<0.05) are indicated by *.

Time-frequency (TF) statistical analysis revealed a sustained power decrease in the alpha band for each of the nine hierarchical sub-conditions compared to the baseline level (Figure 5A and 5B). This decrease was observed in similar locations in the nine contextualized conditions and was maintained from approximately 200 ms to 700 ms after the stimulus onset (Table 2). More precisely, the alpha suppression was significant over several recording sites in occipital and temporo-parietal regions, including P3, Pz, P4, P7, P8, O1, Oz and O2 (Figure 5C.).

**Table 2:** Mean (± *SE*) alpha power (in Microvolts^2^) computed on Pz for the Highest, Middle and Lowest player as a function of the time interval ([100–400 ms] and [400–700 ms]).

	Player's rank							
	Highest		Middle		Lowest		Total	
**Time Window**	M	*SE*	M	*SE*	M	*SE*	M	*SE*
100–400 ms	−523,6	1838,2	−409,4	2234,8	−427,5	1716,5	−453,5	1901,4
400–700 ms	−1082,8	2512,5	−639,3	2755,6	−573,9	2761,1	−765,3	2631,2
**Total**	**−803,2**	**2184,1**	**−524,3**	**2470,7**	**−500,7**	**2262,8**	**−609,4**	**2288,8**

The effect on the alpha response of player rank, participant rank and their interaction was evaluated in the 400-700 ms time window based on the mean alpha profiles in the three player rank conditions and the three participant rank conditions (Figure 5B) using the Wilcoxon test (Figure 5C). The ANOVA performed on the mean alpha signal revealed a main effect of caudality (F(2,30)  =  6,79; *p* < 0.01), indicating that the alpha energy decrease was more pronounced over the parietal domain than the central and frontal domains (*p*< 0.01). A main effect of player rank was also observed on the mean alpha response (F(2,30)  =  3,63; *p* < 0.05), and post-hoc comparisons revealed that highest-rank faces produced the most significant alpha power decrease, compared to middle (*p*< 0.05) or lowest-rank faces (*p*< 0.05) (Figure 5D, see also Figure S2 for scatterplots depicting the pairwise comparisons).

Moreover, neither a significant effect of participant rank (F(2,30)  =  0,75; *p*  =  0.48) nor a player rank × participant rank interaction (F(4,60)  =  0,23; *p*  =  0.92) was observed. It should be noted that similar results were obtained when we performed the same statistical analysis on time windows shifted by ± 50 ms compared to the initial interval.

## Discussion

This study aimed to gain some insight in the temporal resolution of the brain response elicited by faces associated with distinct hierarchical ranks. Three results were found: 1) there was no influence of hierarchy on the amplitude of early components, the occipital P100 and the occipito-temporal N170, 2) highest-rank faces elicited a higher LPP and 3) those faces yielded a stronger alpha suppression. The first result differs from findings of an earlier ERP study that investigated the processing of status conveyed by facial postures [37]. In the latter study, participants were presented with faces with dominant, submissive or neutral expressions and asked to make gender judgments. The authors report that the amplitude of an N200 component, whose topography is similar to that of N170, was largest for dominant faces and smallest for submissive ones. The discrepancy between this observation and the lack of N170 modulation in the current study is most likely due to differences between the two experiment designs. First, head orientation and gaze direction, which were used by Chiao and colleagues to manipulate dominance and submission, can influence facial processing regardless of the expressions they may convey. For instance, several ERP studies have reported a greater N170 amplitude for faces with a direct gaze compared to faces with an averted gaze [55]–[57]. Second, it is not clear to which extent facial expression and gaze orientation convey dominance or submissiveness when the participants are not asked to evaluate hierarchy but instead instructed to make a gender judgment. As an example, a downward gaze orientation could express submission in an explicit hierarchy context, but in the absence of such context, it could simply indicate attentional focus on a downward point in space.

In the current experiment, the absence of N170 effect might simply reflect a null finding and should therefore not necessarily be interpreted as indicating that the hierarchy does not modulate this waveform. It is however worth considering, from methodological and conceptual viewpoints, why we might not have facilitated the observation of an N170 modulation. First, one reason may result from the specific requirements of the task. It has been proposed that task demands are likely to modify face processing [58], [59] and the N170 amplitude [36], [60], [61]. Consequently, it might be that specific task constraints and the hierarchy manipulation of the present experiment interacted in such a way that no modulation on the N170 could be observed. Some authors have proposed that the discrepancy between studies reporting an influence of social category on the N170 amplitude and those reporting no modulation may be accounted for by task demands and stimuli presentation. In particular, they claim that top-down social influences are more likely when 1) the task requires attending to the social dimension investigated [34], [35], [62] and 2) when faces are presented in the context of other faces rather than nonface stimuli [34]. Given that these two criteria were satisfied in the current experiment, top-down hierarchical effects on the N170 should have been facilitated. On the other hand, in contrast with previous studies, which examined only two antagonistic social categories, the present experiment involves *three* categories (i.e. three ranks) and one cannot exclude that this increase in task complexity might have obfuscated the modulation of the N170.

Second, in contrast with some other social categories, such as sex and race, which are also *perceptual* categories, the type of hierarchy investigated here is *purely* social. Yet, perceptual expertise is likely to play a role in modulating the N170 in a social context, and might explain previous reports of a modulation of this component. As observed by Wiese et al. [63], the left-hemispheric N170 effects significantly correlated with the own-race bias, meaning that when participants had greater expertise with other race faces, the N170 amplitude elicited by other-race faces tends to diminish. This result suggests that when a participant has a high perceptual expertise in processing faces from a given social category (e.g. own-race faces) and a low perceptual expertise in the opposite category (e.g. other-race faces), N170 effects are more likely to occur. In the present experiment none of the faces' ranks could be associated with different levels of perceptual expertise: all faces were own-race, own-sex and own-age, and participants were equally presented within the three categories of faces. Furthermore, all faces were neutral and were counterbalanced across participants.

Third, a hierarchical rank is a less stable social category than the ones typically investigated in the field of face processing. In most primate species, social competition typically results in transient outcomes. An individual who occupies the highest rank at a given time will not necessarily do so later. Even more important is the fact that, in the human species, social hierarchies are context dependent; a chess master will not necessarily end up being a kung fu master. In the present experiment, it is very unlikely that participants will view the highest-ranking individuals to prevail in any type of social competition. In contrast, sex, race, and familiarity do not usually change over time and across contexts. Given that the social modulation of the N170 has only been observed with such categories it could be that this modulation cannot be generalized over more tenuous social categories, such as those involved by social hierarchy.

Finally, one can also consider that the type of social hierarchy investigated may determine the incidence of top-down effects on the N170. This echoes with the literature on familiarity which shows that the type of familiar faces affects the N170 amplitude. Several authors report that while famous faces did not elicit a larger N170/M170 amplitude as compared to unknown faces, faces of personal importance to the participant did [29], [31]. In the present experiment, hierarchy is revealed by the level of cognitive performance and is thus related to some form of prestige. However, other forms of prestige, including more qualitative differences (e.g. moral prestige, accomplishing a feat), and other kinds of hierarchy, namely power and dominance, might have a greater social impact and might modulate the N170. Future research should examine this issue.

Although, we did not find any influence of hierarchical status on the N170, we did observe an effect on a later processing stage, revealed by the modulation of a positive potential in the 400–700 ms time-window. Interestingly, as indicated above, such LPP modulations have been repeatedly observed during social categorization of faces. In the present experiment, we observed that highest-rank faces yielded greater LPP amplitude than middle- or lowest-rank faces, regardless of the participant's rank. This effect may be explained by considering that high-status-related information is likely to be more relevant than low-status-related information. A higher status is socially and evolutionarily more desirable than a lower status, and high-status individuals exert a greater social influence. Cognitive resources are thus likely to be allocated to high-status information. In line with this view, it has been observed that people target more of their eye fixations at high-status individuals, indicating a greater level of attention to these individuals [64], [65] and remember them better [66]. They are also more likely to search for information on those individuals than low-ranking individuals especially in high motivational contexts [67]. The LPP has been observed when a large amount of cognitive resources is involved [68] as well as in relation to motivationally relevant pictures that are likely to recruit these resources [52], [54], [69]–[71]. For instance, Schupp et al. [69] report that a threatening face, whose processing is of high evolutionary importance, elicits a late and sustained positive potential over centro-parietal sensors, compared to neutral and friendly faces. The LPP has been largely conceived as indexing the processing of significant stimuli such as arousing pleasant and unpleasant pictures [72]–[74] as well as the evaluative or non-evaluative categorizations associated with affective and attitudinal judgments [75], [76]. Within this framework, the LPP is typically viewed as the activation of motivational systems [54], [71], [77]. In line with this view, the higher LPP amplitude observed for highest-rank faces may thus reflect the recruitment of attentional and motivational resources dedicated to the evaluation of highly significant social stimuli.

Another result in line with the interpretation that the LPP indexes attentional and motivational effects is the greater LPP amplitude for the lowest rank faces compared to the middle rank faces. Lowest rank faces refer to individuals who occupy the worst position in the hierarchy. Generally speaking, the low-end of a social scale is of critical importance. Not only does a low social rank decrease reproductive success [5], [6], but in stable hierarchies, it also results in greater stress and in poorer health [78]. Hence, tracking the low-end of a social scale might be more valuable than tracking the middle part of the scale so as lowest rank faces might be cognitively and emotionally more significant than middle rank faces.

At a more mechanistic level, a key issue worth being addressed relates to the brain structures generating and modulating the LPP reported here. Interestingly, recent studies have tried to identify the structures generating and modulating the LPP elicited by emotional pictures [79]–[81]. This work consists in combining EEG and fMRI techniques and analyzing the relationship between the EEG and BOLD signals. Those studies reported that the LPP amplitude correlated with BOLD activity in visual cortical structures but also in corticolimbic and subcortical structures, including the amygdala and insula (see [80], [81]). These results have been interpreted in line with the reentrant feedback hypothesis which stipulates that in the context of motivationally relevant stimuli, deep structures, such as the amygdala, and cortical structures modulate the visual cortices by reentrant feedback [80], [81].

The activation of amygdala in those studies should be related with the work of Kumaran et al. [12] which focuses on the representation of social and non-social hierarchies. These authors observed that the neural activity in the amygdala specifically correlated with the knowledge of a social hierarchy and also correlated with the motivational value associated with individuals according to their social rank. Interestingly, the activation of amygdala has also been reported in the context of unstable hierarchies, in which the rank of participants was moved up and moved down during a game phase [9]. In particular, viewing a high-rank player resulted in greater activity in the right amygdala [9], (see also [82]).

Following the perspective of this work, which links activity in the amygdala with the processing of highly motivational stimuli, it might be worth investigating in the future whether the LPP amplitude found in a hierarchical context correlates with the neural activity in the amygdala. Indeed, in the current experiment, participants were directly embedded in the hierarchy and consequently ranks clearly established distinct social values, with the highest rank being of highest value. Of course, other structures might also be implicated in the LPP modulation we observed, and could include regions specifically involved in the processing of social and hierarchical stimuli, such as the dorsolateral prefrontal cortex [9], [83], the ventrolateral prefrontal cortex [10], [11], or regions involved in value comparison, such as the ventromedial prefrontal cortex [84], the latter being also known to correlate with the LPP amplitude [81].

To more precisely determine the nature of the processes involved in status categorization, we also investigated alpha oscillations, which have previously been shown to be modulated by face processing [40], [41]. Our findings indicate that while all faces elicited a significant alpha desynchronization, the extent of the desynchronization depended on the absolute rank of the face. Highest-rank faces were associated with stronger alpha suppression. This effect was observed irrespective of the relative position of the participant and was maximal approximately 400 ms after the face onset. Synchronization in the alpha-band has traditionally been interpreted as a marker of cortical idling, but recent approaches consider the role of alpha oscillations to be related to functional inhibition (see [85] for a review) and cortical excitability [86]. Indeed, the oscillatory pattern is often characterized by a decrease of alpha activity in cortical regions specifically involved in the task and by an increase of alpha activity in task-irrelevant regions. Therefore, alpha synchronization can be seen as a way to neutralize task-irrelevant regions [85], while alpha desynchronization would indicate a greater engagement of the regions affected by this suppression. According to this view, greater alpha suppression observed for the highest-rank faces would reflect a greater allocation of cognitive resources to these stimuli. This hypothesis is perfectly in line with the above cited findings indicating that high-status-related stimuli tend to capture more of our cognitive resources.

Interestingly, alpha suppression has also been reported for another type of social categorization, namely familiarity [41]. Zion and colleagues observed that at occipital sites in the 200–800 ms time window, the alpha suppression was stronger for famous faces than for unfamiliar faces. The authors attribute this effect to the use of semantic knowledge associated with faces. However, in the present experiment, the amount of semantic information associated with a face is similar for the three hierarchical conditions. It is rather the relevance of face-associated ranks that differs among the conditions: the highest-rank position is the most relevant and should attract attention more. This is consistent with a wide range of studies showing that alpha suppression is associated with attention [87]–[92].

Taken together the LPP and alpha oscillations results showed that the only experimental variable which significantly modulated the brain response was player rank. Participant rank had no effect and did not interact with player rank. Hence, no matter the participant's position in the hierarchy, player's faces elicited similar brain responses. For instance, this means that when participants had a high or a low social rank, viewing the face of the highest player in the hierarchy resulted in a similar effect. Yet, we could have expected that when participants are in a low social position, viewing a high-rank face would have a greater impact than when they are in a high social position. Indeed, in a low social position, the faces of highest rank players might elicit a greater motivational relevance as they refer to players who achieved a much better level of performance than that of participants'. In contrast, when participants are in the highest social position, high-rank faces might have a weaker social value. Overall, this pattern of results suggests that the brain response is more sensitive to the faces' absolute ranks than to the social distance between the participant's rank and the player's rank. However, it should be noted that in the current experiment, participants' social position was temporary and changing (it was counterbalanced across the highest, middle and lowest ranks). It follows that the social distance between the participants and the other players was not a very stable variable. This contrasts with standard human organizations in which people ranks last for much longer time. In such contexts, the social distance is a less noisy variable and is therefore more likely to influence cognitive processing. Future work could thus investigate whether stable social distance modulates face processing in such ecological organizations.

In conclusion, this study provides new insight into the neural processing underlying status perception and, at a broader level, social categorization. Most electrophysiological studies addressing the social categorization of faces investigate features that are often not purely social because they include encyclopedic knowledge (for famous faces) or physical differences [13]. In the present work, social rank was manipulated without being associated with a particular physical posture so that each face could be assigned to each of the three ranks explored. It is also worth noting that many studies that explore the impact of familiarity on face processing use well-known politicians and artists to manipulate fame, which is obviously a mark of prestige and high status. It is possible that the similar pattern of results between these studies and ours may originate from the common social parameter that serves to categorize faces, namely status. Overall, our results are consistent with the view that adaptive monitoring of our social environment requires enhanced tracking of the upper end of the social scale but of course further work is needed to determine whether the effects reported here reflect the involvement of hierarchy-specific mechanisms or more general mechanisms.

## Supporting Information

Figure S1 **Scatterplots depicting the pairwise comparisons of LPP amplitude for the three players rank conditions.** Top left: highest vs middle, top right: highest vs lowest, bottom: middle vs lowest. Values were computed on the central scalp region (FZ, CZ, PZ) where the effect was significant. Each dot refers to one particular participant. This particular LPP pattern was observed for a large proportion of subjects: in the midline region, 75% of them displayed a larger LPP for the highest player compared to the middle one, 62,5% compared to the lowest one, and 81,25% of them showed a larger LPP for the lowest player compared to the middle one.
(DOC)

Figure S2 **Scatterplots depicting the pairwise comparisons of averaged alpha power for the three player rank conditions.** Top left: highest vs middle, top right: highest vs lowest, bottom: middle vs lowest. Values were computed on Pz in [400–700 ms] where the effect was significant. Each dot refers to one particular participant.
(DOC)
