# Supplementary figures and images for: Face the Hierarchy: ERP and Oscillatory Brain Responses in Social Rank Processing
Source: PLoS One. 2014 Mar 12;9(3):e91451. doi: 10.1371/journal.pone.0091451 (PMC3951356; doi:10.1371/journal.pone.0091451)

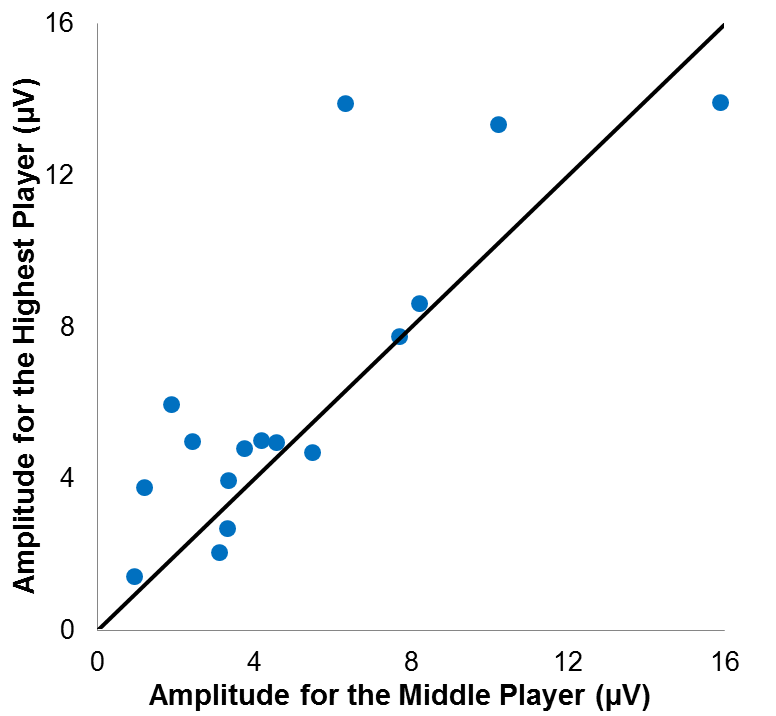

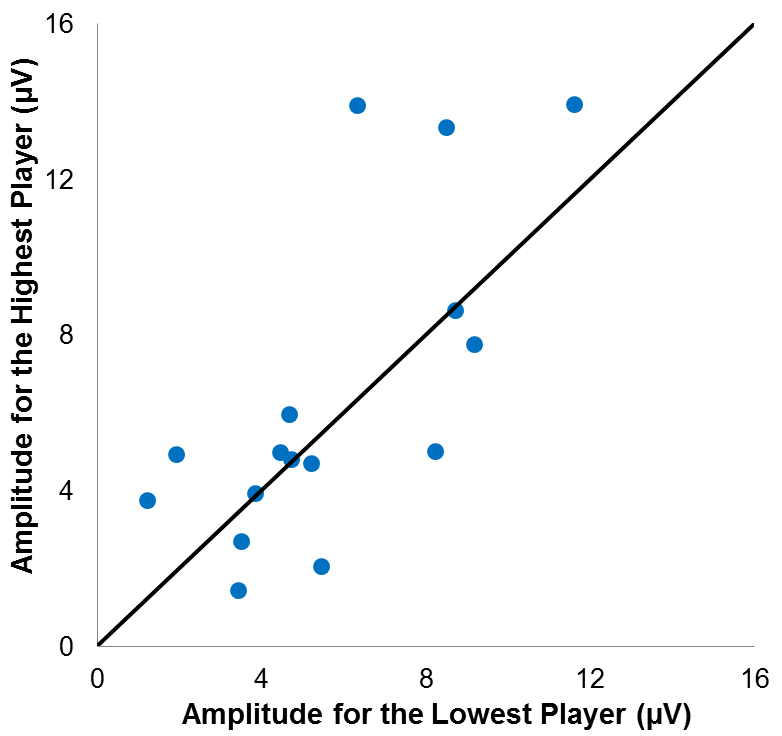


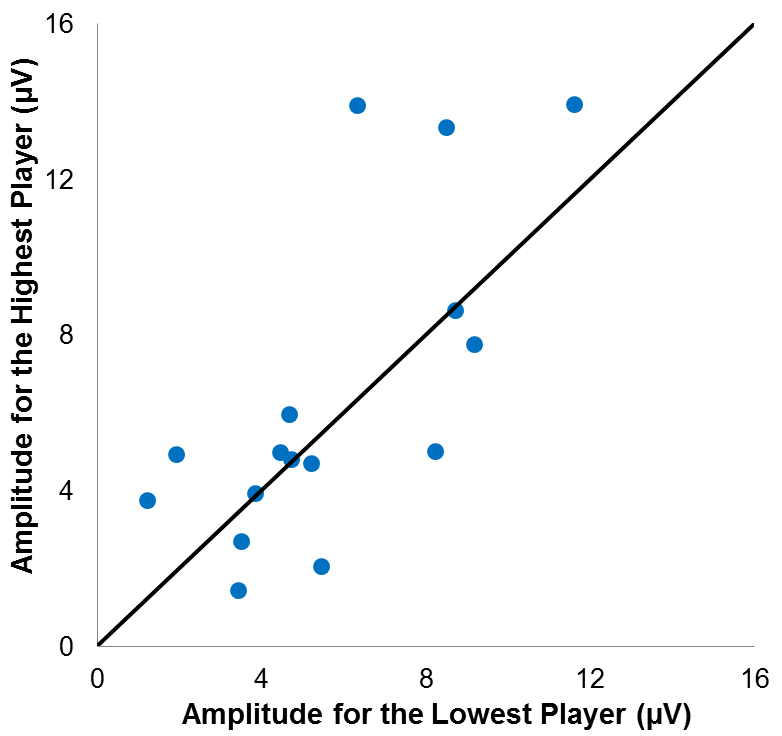

Supplement: Figure S1 — Scatterplots depicting the pairwise comparisons of LPP amplitude for the three players rank conditions. Top left: highest vs middle, top right: highest vs lowest, bottom: middle vs lowest. Values were computed on the central scalp region (FZ, CZ, PZ) where the effect was significant. Each dot refers to one particular participant. This particular LPP pattern was observed for a large proportion of subjects: in the midline region, 75% of them displayed a larger LPP for the highest player compared to the middle one, 62,5% compared to the lowest one, and 81,25% of them showed a larger LPP for the lowest player compared to the middle one. (DOC) [file pone.0091451.s001.doc]

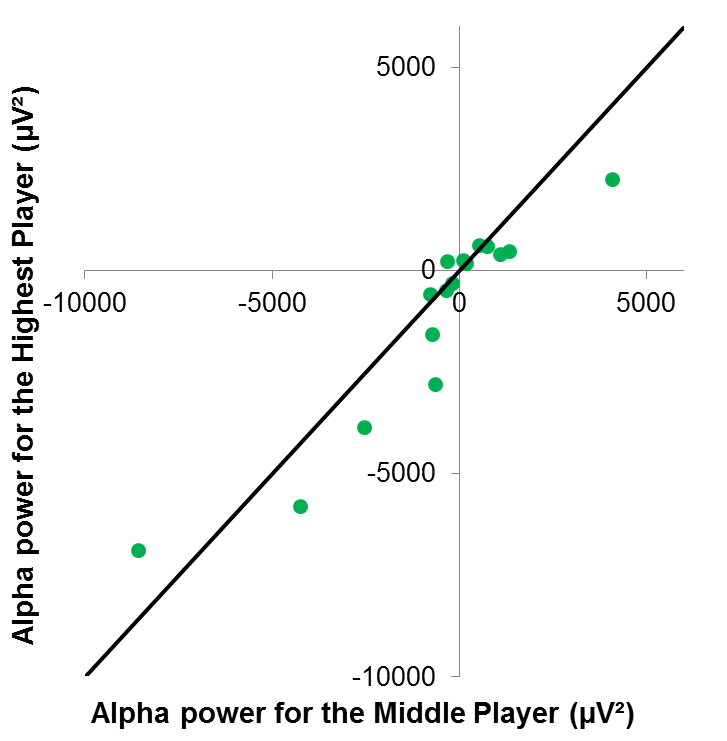

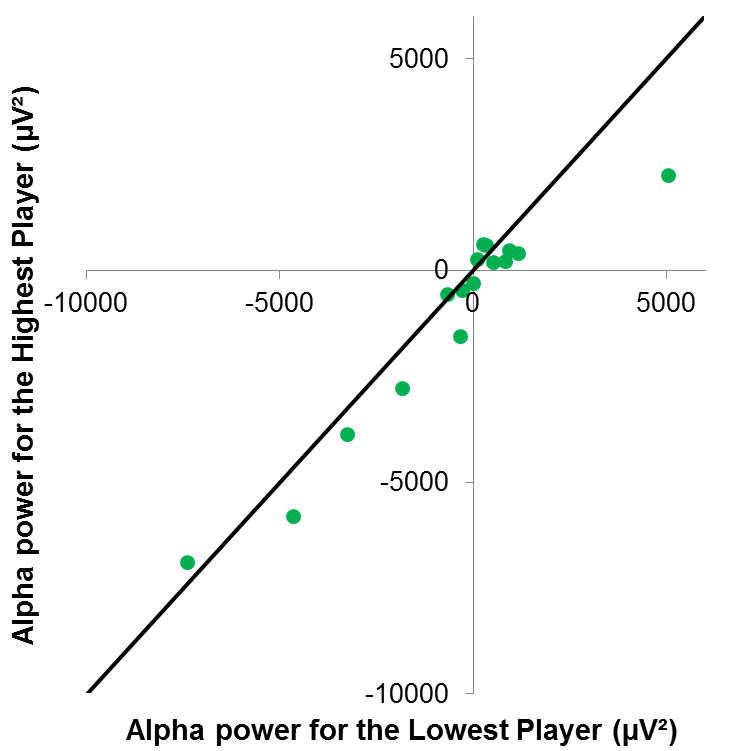


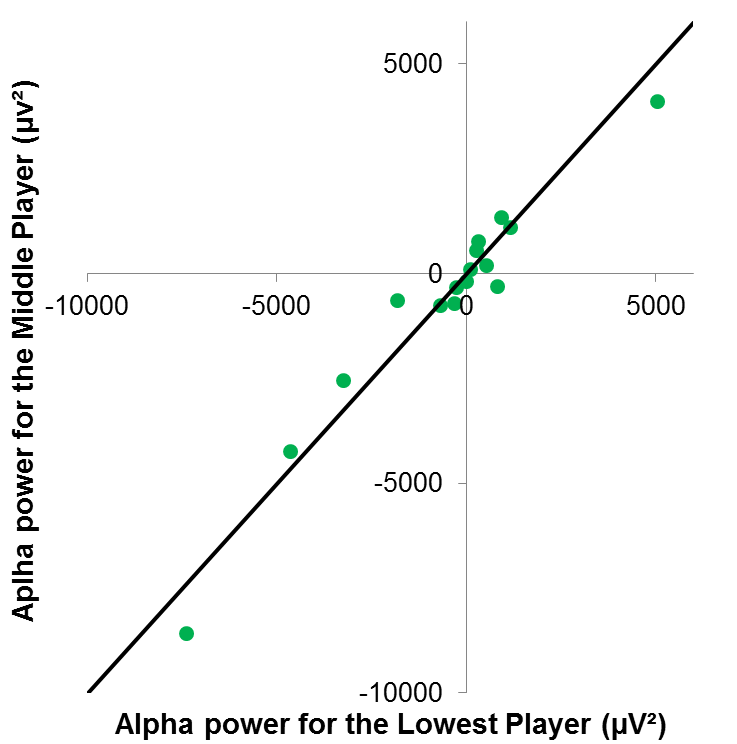

Supplement: Figure S2 — Scatterplots depicting the pairwise comparisons of averaged alpha power for the three player rank conditions. Top left: highest vs middle, top right: highest vs lowest, bottom: middle vs lowest. Values were computed on Pz in [400–700 ms] where the effect was significant. Each dot refers to one particular participant. (DOC) [file pone.0091451.s002.doc]
